# P18 Comparing paediatric OPAT and hospital IV/intramuscular (IM) antimicrobial use: a matched cohort analysis from Oxford, UK

**DOI:** 10.1093/jacamr/dlaf239.022

**Published:** 2026-01-05

**Authors:** Givani Amarakoon, Christina Newbould, Alice Payne, Georgina Thorne, Leila Willis, Nicole Hobson, Rebecca Nip, Laura Wilkins, Stephane Paulus, Zoe Rooney

**Affiliations:** Oxford University Hospitals NHS Foundation Trust, Oxford, UK; Oxford Health NHS Foundation Trust, Oxford, UK; Oxford Health NHS Foundation Trust, Oxford, UK; Oxford Health NHS Foundation Trust, Oxford, UK; Oxford Health NHS Foundation Trust, Oxford, UK; Oxford Health NHS Foundation Trust, Oxford, UK; Oxford University Hospitals NHS Foundation Trust, Oxford, UK; Oxford University Hospitals NHS Foundation Trust, Oxford, UK; Oxford University Hospitals NHS Foundation Trust, Oxford, UK; Oxford University Hospitals NHS Foundation Trust, Oxford, UK

## Abstract

**Objectives:**

To evaluate the clinical characteristics, spectrum of antimicrobial use, and complication rates among children receiving outpatient parenteral antimicrobial therapy (OPAT) at a large UK centre, and to compare these with matched hospital inpatients treated with IV/IM antimicrobials for similar infectious indications.

**Methods:**

We conducted a retrospective review of paediatric OPAT episodes from August 2024 to June 2025, including all cases where children received IV or intramuscular antimicrobials. Each OPAT referral was matched to two hospital inpatients by indication and age band, using a comprehensive hospital dataset of IV/IM antimicrobial administrations during the same period. Data collected included demographics, antimicrobial class, route of administration, number of doses or therapy duration, indication, and (for OPAT only) treatment complications.

**Results:**

Forty paediatric OPAT episodes (median age 3 years, 60% female, 90% from John Radcliffe Hospital, 10% from Horton Hospital) were compared to 78 matched hospital inpatients (median age 2 years, 92% from John Radcliffe, 8% from Horton Hospital). The OPAT cohort included 37 antibiotic courses (92.5%), 2 antiviral (5%), and 1 antifungal (2.5%). The most commonly used antimicrobials in OPAT were ceftriaxone, caspofungin, and ertapenem, while ceftriaxone, co-amoxiclav, and ceftazidime predominated among hospital inpatients. The median number of days in OPAT was 3.0, with a mean of 3.3 days. For hospital inpatients, the median therapy duration was 5.0 days, with a mean of 8.5 days. In terms of route, In the OPAT cohort, IV access was most commonly via peripheral cannula (70%), followed by midline (10%), Hickman line (7.5%), and PICC line (5%). Intramuscular administration was used in 10% of episodes, including one patient who received both midline and IM therapy. By contrast, 96.2% of hospital courses used IV administration, predominantly via peripheral cannula or central line, and 3.8% by intramuscular route. The main indications in OPAT were urosepsis and presumed sepsis, while urosepsis and lower respiratory tract infection were most frequent in the matched hospital group. Treatment-related complications occurred in 17.5% (7/40) of OPAT episodes, primarily due to peripheral cannula issues (tissuing, failure, or leakage) necessitating change in access or early therapy adjustment. All complications were managed without serious adverse outcomes, with most patients completing their course via alternative routes or oral switch.

**Conclusions:**

Paediatric OPAT in Oxford, delivered as part of our integrated Hospital at Home service, provides focused antimicrobial therapy predominantly via peripheral IV access, with smaller proportions using midlines, PICC lines, or Hickman lines, and a notable proportion administered intramuscularly. The antimicrobial spectrum in OPAT is narrower than in general inpatient practice, reflecting targeted use for clearly defined infectious indications. Line-related complications occurred in nearly one-fifth of episodes, but all were manageable without serious adverse outcomes. At present, Hospital at Home OPAT patients are not routinely reviewed on antimicrobial stewardship ward rounds, highlighting a clear opportunity to embed formal AMS oversight into the service to further enhance governance, optimize antimicrobial use and benchmark outcomes.

